# Editorial: Child Sexual Abuse: Empirical Research on Understanding and Helping Victims and Offenders

**DOI:** 10.3389/fpsyg.2022.844639

**Published:** 2022-02-16

**Authors:** Taina Laajasalo, Noora Ellonen, Robert Horselenberg, Cristina Izura, Nadia Wager

**Affiliations:** ^1^Special Services, National Institute for Health and Welfare, Helsinki, Finland; ^2^Department of Psychology, University of Helsinki, Helsinki, Finland; ^3^Faculty of Social Science, University of Tampere, Tampere, Finland; ^4^Department of Criminal Law and Criminology, Faculty of Law, Maastricht University, Maastricht, Netherlands; ^5^Department of Psychology, Swansea University, Swansea, United Kingdom; ^6^Department of Behavioural and Social Sciences, University of Huddersfield, Huddersfield, United Kingdom

**Keywords:** child sexual abuse, editorial, prevention, victimization, offending, violence - prevention and control

Child sexual abuse (CSA) is a global problem with multiple long-term adverse effects and public health consequences. In a recent umbrella review, CSA was associated with elevated risks of various negative long-term psychosocial, psychiatric, and physical health outcomes, and the population attributable risk fractions for several common mental health problems were considerable (Hailes et al., 2019). Early findings from the COVID-19 era are causing growing concerns about elevated risks and rates of CSA (Fore, 2021). Therefore, the subject of this Research Topic is ever so important. The aim was to create a multidisciplinary compilation of latest research findings on issues directly or indirectly related to CSA. This aim was achieved: a total of 13 articles from several disciplines address CSA from multiple theoretical and methodological viewpoints. Prevention of CSA and its consequences demands taking into consideration the perspectives and needs of victims and potential victims, perpetrators and potential perpetrators, criminal justice system, social and health care services as well as the context, culture, and society as a whole. The importance of this comprehensive outlook is highlighted by the articles in this issue.

In this issue, three articles (Erens et al.; Goodman-Delahunty et al.; Magnusson et al.) bring forward important topics related to criminal justice system. These are essential in getting CSA cases investigated, prosecuted and convicted, simultaneously securing the rights of the victims as well as the defendants. First, Erens et al. show how many professionals conducting CSA assessments within the child protective services still endorse beliefs not in line with scientific evidence, and often do not use interviewing methods with empirical support. Magnusson et al. find associations between Nordic police interviewers' self-reported goals, tactics, and emotions during CSA suspect interviews. For example, interviewers experiencing more negative emotions, such as anger, were more likely to employ confrontational tactics, such as focusing on obtaining a confession or utilizing aggressive tactics like raising one's voice. Further, Goodman-Delahunty et al. show that educating jurors in the form of judicial direction and expert evidence from a psychologist significantly increases jurors' CSA knowledge, which in turn enhances the credibility of the complainant and increases the conviction rate. As a whole, these articles underline the importance of training various officials working within the CPS and the justice system, and how these efforts may aid in securing access to justice for all parties. Further, educating needs to extend to potential victims too: May et al. show how attending a theater-in-education program on CSA helped participants gain new awareness and understanding of aspects related to child sexual exploitation and abuse. Results from a total of four focus groups tentatively indicate an increase in young people's awareness and knowledge around CSA, including victims, perpetrators and abusive relationships, as well as how to avoid harm and maintain safety. Future research is needed to see whether training will translate into actual behavioral change, such as increases in reporting and disclosures of CSA.

The focus of public policies and interventions on CSA perpetrators is still mostly on measures that take effect after the offense has taken place. As an example, risk assessment measures have been developed for correctional settings, limiting their utility with non-offending, at-risk individuals. An important prevention strategy would be the utilization of validated risk assessment tools, addressing especially dynamic, potentially changeable risk factors among potential perpetrators. To this end, Wittström et al. address the risk of CSA among 55 non-correctional patients with pedophilic disorder, which thus far is largely unknown. Measuring research-based dynamic risk factors and self-rated risk of CSA among self-referred, help-seeking men, they found that these participants scored higher than controls (age-matched non-clinical control group) on all these domains. In addition, Lampalzer et al. assess how the acceptance and integration of the sexual preference into the individual self-concept is associated with pedophilia associated urges and behaviors among help-seeking individuals. Findings suggest that more acceptance of ones' sexual preference might lead to both negative behaviors (the frequency of sexual activities with minors) and positive behaviors (the frequency of use of legal imagery of children) in relation to treatment goals, highlighting the need for further research and individualized treatment plans. Finally, an article by Ferretti et al. compares sexual offenders (76% of whom were child molesters) to other offenders with heterogeneous criminal histories, but without sex offenses, utilizing validated risk assessment measures (HCR-20 V3). They show the role of deficient non-intimate relationships as a risk factor for criminal careers of sex offenders. Sexual offenders had a smaller likelihood of history of problems with violence and antisocial behavior than other offenders, but were more likely to have problems in non-intimate relationships with family and friends. In addition, interpersonal and affective deficits measured on the Psychopathy Checklist-Revised (PCL-R) boosted this association: when problems with non-intimate relationships were possibly or certainly present, these psychopathic traits increased the likelihood of being a sex offender as compared with other offenders. Although questions of causality remain, these findings is important in terms of developing and tailoring treatment.

Regarding victims and potential victims, the heterogeneity of individuals and their experiences must be acknowledged. Jones highlights the contextual specificities in child sexual abuse regarding Caribbean women and their CSA experiences, and also addresses the links between intimate partner violence and violence against girls. Further, in vulnerable circumstances where patriarchal values, structural disadvantage and interpersonal violence are commonplace, interconnected contextual risks of CSA can be identified. These include the prevalence of early sexualization of children and the social acceptance and attitudes contributing to sexual violence. Heino et al. show that transgender identity and non-binary identity are associated with both being bullied and bullying others even when controlling for a range of variables. As the authors note, it is known that a large proportion of bullying among adolescents is of a sexual nature, and thus these findings are relevant to the prevention of CSA experiences. Schoon and Briken point out survivors' obstacles in dealing with CSA, utilizing data from Independent Inquiry Into Child Sexual Abuse in Germany. Many of the experienced obstacles were linked to the criminal justice system and mental health services, such as the duration of judicial proceedings or lack of access to psychotherapy, providing guidance on how our service system can avoid re-traumatizing the victim and instead aid the victim in regaining control.

Digital development and internet communication have fundamentally changed the field of CSA. Battling technological-assisted CSA requires better understanding of this evolving phenomenon. In their systematic review and economic approach, Giles and Alison underscore the large scale of the problem and its huge economic burden, which amounts to billions in the UK alone. This translates to requirements of research-informed prioritization by the police, where the offenders of highest risk downloading and sharing incident images of children should be identified and targeted first. Further, Woodhams et al. posit how the use of the Dark Web for purposes related to CSA is growing and analyze data from 53 anonymous suspected abusers. They described the suspects' characteristics, and their motivations for using the Dark Web, knowledge that may be of use when targeting offenders. Finally, Joleby et al. bring into attention the victims of technology-assisted CSA. Based on in-depth interviews of seven young women they show how victimization has influenced their well-being profoundly, both immediately and in the long term and how it has changed how they perceive themselves and their relations to other people.

The road to evidence-based responses to CSA is not an easy one. The breadth of the Research Topics in this special issue tell of the complexity surrounding CSA. The only way to effectively prevent sexual violence is to adopt a comprehensive strategy consisting of primary, secondary and tertiary preventive measures. This is also required by international treaties and agreements such as the Council of Europe Convention on Protection of Children against Sexual Exploitation and Sexual Abuse. We hope that this special issue will contribute to the development and implementation of evidence-based prevention strategies and will encourage both researchers and practitioners to continue their efforts to eradicate CSA.

## Author Contributions

TL and NE wrote the editorial. TL, NE, RH, CI, and NW contributed to conception of the editorial and approved the submission. All authors contributed to the article and approved the submitted version.

## Conflict of Interest

The authors declare that the research was conducted in the absence of any commercial or financial relationships that could be construed as a potential conflict of interest.

## Publisher's Note

All claims expressed in this article are solely those of the authors and do not necessarily represent those of their affiliated organizations, or those of the publisher, the editors and the reviewers. Any product that may be evaluated in this article, or claim that may be made by its manufacturer, is not guaranteed or endorsed by the publisher.
